# Androgen and glucocorticoid crosstalk in granulosa cells: molecular insights into polyendocrine metabolic ovarian syndrome

**DOI:** 10.3389/fendo.2026.1943976

**Published:** 2026-09-24

**Authors:** Martina Pinna, Alessandra Tomaselli, Davide Ferrari, Marianna Minnetti, Daniele Gianfrilli, Elisa Giannetta, Carlotta Pozza, Andrea M. Isidori, Federica Barbagallo, Riccardo Pofi

**Affiliations:** 1 Department of Experimental Medicine, Sapienza University of Rome, Rome, Italy; 2 Department of Medicine and Surgery, “Kore” University of Enna, Enna, Italy; 3 Oxford Centre for Diabetes, Endocrinology and Metabolism, National Institute for Health and Care Research (NIHR) Oxford Biomedical Research Centre, University of Oxford, Churchill Hospital, Oxford, United Kingdom

**Keywords:** 11-oxygenated androgens, 11β-HSD, androgen receptor, folliculogenesis, glucocorticoid receptor, granulosa cells, polycystic ovary syndrome

## Abstract

Polyendocrine metabolic ovarian syndrome (PMOS), previously named polycystic ovary syndrome (PCOS) is a multifaceted endocrine-metabolic disorder characterized by hyperandrogenism, ovulatory dysfunction, and polycystic ovarian morphology. At the cellular level, impaired folliculogenesis represents a key feature, with granulosa cells failing to optimally support follicle maturation, steroidogenesis, and oocyte competence. This review evaluates the hypothesis that granulosa cell dysfunction in PMOS may, in selected phenotypes, be prompted by altered molecular crosstalk between the androgen receptor and the glucocorticoid receptor. These two nuclear receptors integrate reproductive, metabolic, and stress-related signals within the ovarian microenvironment. Because androgen and glucocorticoid receptors share partially overlapping DNA-binding motifs and co-regulatory networks, their interaction may shape transcriptional programs governing proliferation, steroidogenesis, mitochondrial metabolism, and stress adaptation. Emerging evidence also highlights the contribution of adrenal-derived 11-oxygenated androgens, potent androgen receptor agonists, which amplify androgen signaling and reinforce the hyperandrogenic milieu. By integrating evidence from molecular, experimental, and patient-derived studies, this review proposes androgen and glucocorticoid receptor crosstalk as a plausible and testable regulatory framework, in PMOS pathophysiology with potential implications for biomarker discovery and future mechanism-based therapeutic strategies.

## 1 Introduction

Polyendocrine metabolic ovarian syndrome (PMOS), previously named polycystic ovary syndrome (PCOS), was first described in 1935 by Stein and Leventhal as a condition characterized by hirsutism, amenorrhea, chronic anovulation, infertility, obesity, and enlarged polycystic ovaries (1). It has since been recognized as one of the most common endocrine disorders affecting women of reproductive age. Estimated prevalence ranges from 4% to 21%, varying substantially with diagnostic criteria, ethnicity, age, and recruitment setting (2). Polycystic ovarian morphology, defined by enlarged ovaries containing multiple small antral follicles and increased stromal volume, remains one of the syndrome’s most recognizable features (3–5). Yet morphology alone does not capture its full complexity: many affected individuals present clinical and biochemical abnormalities that extend well beyond the ovary. Following a global multistep consensus process, PMOS was adopted in May 2026 as the new name for PCOS (6). This change concerns nomenclature rather than diagnostic criteria, and its implementation in clinical and research practice is ongoing during this transition period. Accordingly, we use PCOS when referring to studies conducted before this nomenclature change and PMOS/PCOS when referring to the condition more broadly. Clinically, PCOS encompasses multiple phenotypes (**Figure 1**), with variable combinations of hyperandrogenism, oligo-anovulation, polycystic ovarian morphology, insulin resistance, adiposity, dyslipidemia, hypertension, impaired glucose metabolism, and psychological comorbidities such as anxiety, depression, reduced quality of life, and eating disorders (7, 8). This heterogeneity has direct therapeutic implications, since current management remains largely phenotype-driven and symptom-oriented: combined oral contraceptives are commonly used to control menstrual irregularity and hyperandrogenic symptoms; lifestyle interventions and metformin mainly target metabolic features; anti-androgens may be added in selected cases; and letrozole is recommended as first-line pharmacological ovulation induction in anovulatory infertility (7, 9–12). None of these interventions, however, fully addresses the complex and interrelated mechanisms underlying PCOS, and few therapies can currently be considered disease-modifying or specifically targeted to the molecular drivers of ovarian dysfunction. This therapeutic gap reflects the multifactorial nature of PCOS pathophysiology, which arises from the interplay of neuroendocrine dysregulation, insulin resistance, altered steroid hormone signaling, and intra-ovarian dysfunction. Perturbations in the hypothalamic–pituitary axes that regulate reproductive and stress-related responses shape the endocrine environment characteristic of PCOS, influencing gonadotropin secretion, androgen production, and follicular development. Within the ovary, granulosa cells (GRCs) act as key integrators of endocrine, metabolic, and local regulatory signals, coordinating follicular growth, steroidogenesis, and oocyte maturation (13). Emerging evidence points to dysregulated signaling through steroid hormone receptors, notably the androgen receptor (AR) and glucocorticoid receptor (GR), as a potential contributor to GRC dysfunction and impaired folliculogenesis in PCOS (14). Clarifying how these pathways may interact within the ovarian microenvironment could provide new mechanistic insight and identify priorities for future investigation. This review focuses specifically on molecular mechanisms at the granulosa-cell level, particularly AR and GR signaling, as one component of the broader PCOS pathophysiological network; other drivers, while equally important, lie beyond its scope and may warrant dedicated attention in future work.

**Figure 1:**
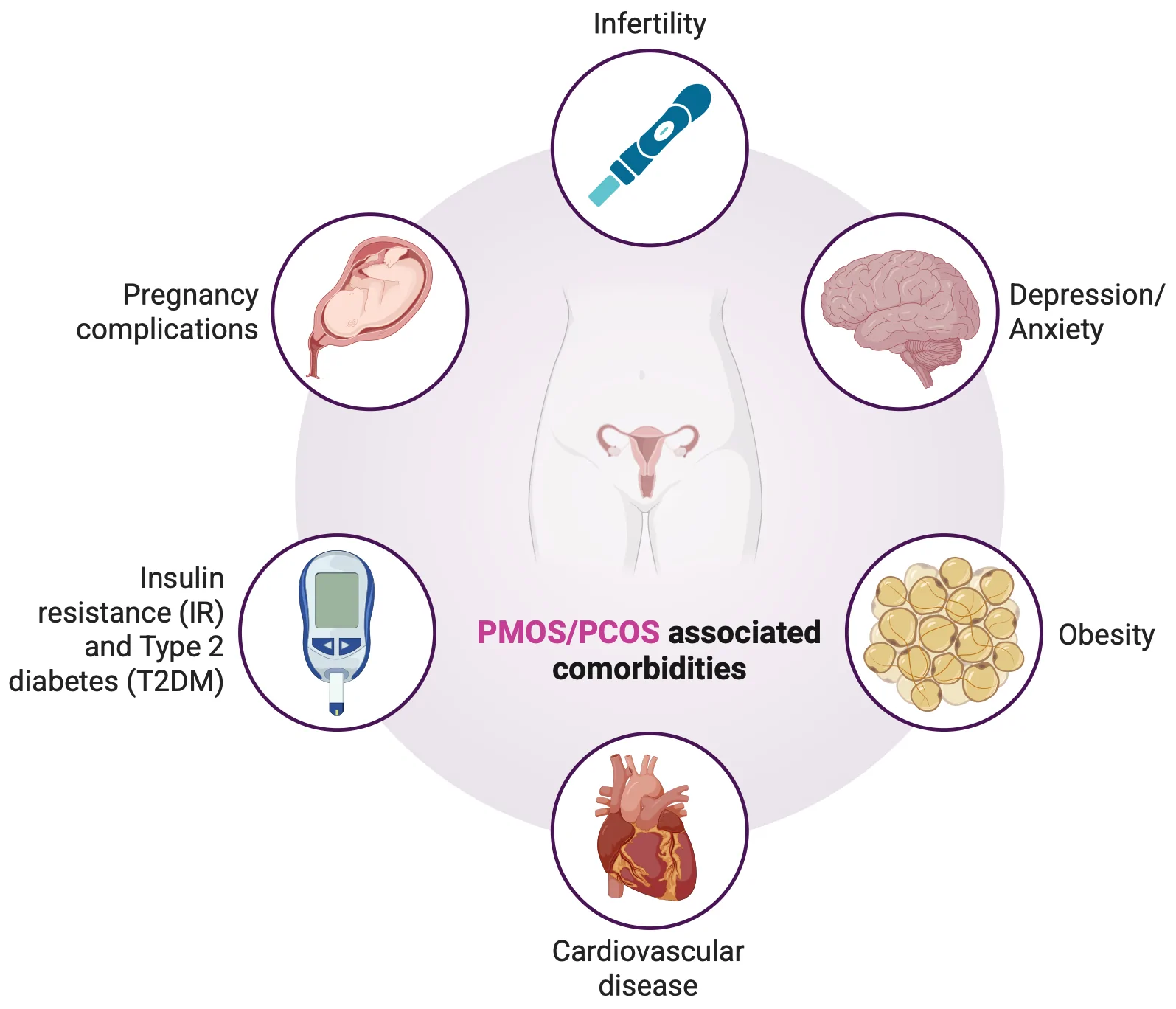
Clinical spectrum of PMOS/PCOS associated comorbidities. Polyendocrine metabolic ovarian syndrome (PMOS), formerly known as polycystic ovary syndrome (PCOS), is associated with a broad spectrum of reproductive, metabolic, cardiovascular, and psychological comorbidities. In addition to infertility and pregnancy complications, individuals with PMOS/PCOS are at increased risk of obesity, type 2 diabetes mellitus (T2DM), cardiovascular disease, and mood disorders, including depression and anxiety. Created in BioRender. Pinna, M (2026). https://BioRender.com/7yom8ck.

## 2 Neuroendocrine regulation in PCOS

### 2.1 Hypothalamic–pituitary–adrenal axis

The hypothalamic–pituitary–adrenal (HPA) axis coordinates stress responses while maintaining metabolic, immune, and endocrine homeostasis (15, 16). Stress-related and circadian inputs converge on paraventricular hypothalamic neurons, which release corticotropin-releasing hormone (CRH), often in combination with arginine vasopressin. These neuropeptides stimulate pituitary corticotroph cells to secrete adrenocorticotropic hormone (ACTH), which in turn promotes glucocorticoid synthesis and secretion by the adrenal cortex, predominantly in the form of cortisol in humans. Cortisol exerts negative feedback at both hypothalamic and pituitary levels through activation of the glucocorticoid receptor (GR; NR3C1), thereby regulating basal activity and stress responsiveness of the HPA axis. A distinctive feature of HPA physiology is its temporal organization. Cortisol secretion follows a robust circadian rhythm, characterized by peak levels in the early morning and a nadir during the night, superimposed on an ultradian pulsatile pattern that contributes to tissue-specific glucocorticoid responses (17). These oscillatory dynamics influence GR activation, chromatin occupancy, and downstream transcriptional programs involved in metabolism, inflammation, and reproductive function. Accordingly, disruption of cortisol rhythmicity is a hallmark of several endocrine disorders, whereas restoration of physiological cortisol dynamics is associated with significant clinical benefits (18–21). Beyond glucocorticoid production, the adrenal cortex also contributes substantially to circulating androgen levels through the secretion of DHEA, DHEAS, and androstenedione under ACTH regulation. In PCOS, a considerable proportion of patients exhibit functional adrenal hyperandrogenism, characterized by increased adrenal androgen production and/or enhanced adrenal responsiveness to ACTH (22). Recent attention has focused on 11-oxygenated androgens (11-OH androgens), a class of adrenal-derived C19 steroids that includes potent androgen receptor agonists such as 11-ketotestosterone. These steroids are frequently elevated in PCOS and may represent a major source of circulating androgenic activity, further strengthening the mechanistic link between adrenal dysfunction and ovarian pathology (23, 24).

### 2.2 Hypothalamic–pituitary–ovarian axis

Beyond the HPA axis, reproductive endocrine function in females is orchestrated by the hypothalamic–pituitary–ovarian (HPO) axis, a highly coordinated neuroendocrine network that controls follicular development, steroidogenesis, ovulation, and fertility (25, 26). At the hypothalamic level, specialized neurons release GnRH in a pulsatile manner into the hypophyseal portal circulation, stimulating anterior pituitary gonadotrophs to synthesize and secrete LH and FSH (27). GnRH pulse frequency and amplitude critically determine the relative secretion of these gonadotropins (28). Within the ovary, LH primarily acts on theca cells, stimulating androgen production, while FSH acts on granulosa cells to promote follicular growth, differentiation, and estrogen biosynthesis (29). According to the classical two-cell, two-gonadotropin model, androgens synthesized by theca cells serve as substrates for aromatization within granulosa cells, ultimately leading to estradiol production (30). Ovarian steroid hormones regulate the HPO axis through feedback mechanisms. During most of the cycle, estradiol exerts negative feedback on GnRH secretion through the KNDy neuronal network (31). During the late follicular phase, sustained estradiol elevation triggers positive feedback, leading to the preovulatory LH surge required for ovulation (32). Following ovulation, progesterone produced by the corpus luteum suppresses GnRH pulse frequency and stabilizes the luteal phase (33, 34).

Neuroendocrine dysfunction of the HPO axis is a well described component of PCOS pathophysiology (**Figure 2**) although its magnitude varies across phenotypes. One of the most consistent endocrine abnormalities is an increase in GnRH pulse frequency, estimated to be approximately 1.3–1.5-fold higher than physiological levels in selected cohorts (35). Growing evidence implicates dysfunction of hypothalamic KNDy neurons. Prenatal androgen exposure and chronic hyperandrogenemia may enhance AR signaling, reduce progesterone receptor (PR) and dynorphin expression, and increase excitatory kisspeptin and neurokinin B signaling, thereby promoting persistent activation of the GnRH pulse generator (36, 37). This dysregulation is further exacerbated by reduced sensitivity to estradiol and progesterone negative feedback. Sustained acceleration of GnRH pulsatility favors LH secretion over FSH release and contributes to the characteristic elevation of the LH/FSH ratio often observed in PCOS (38, 39). The resulting gonadotropin imbalance promotes excessive androgen production by theca cells while simultaneously impairing granulosa-cell differentiation and aromatase expression through relative FSH insufficiency. Reduced FSH signaling diminishes CYP19A1 transcription and estradiol synthesis, favoring intrafollicular androgen accumulation and perpetuating hyperandrogenism (40). Impaired follicular maturation also leads to the accumulation of small antral follicles and increased production of anti-Müllerian hormone (AMH). Elevated AMH further reduces follicular responsiveness to FSH, reinforcing follicular arrest and contributing to endocrine dysfunction (41). Collectively, these neuroendocrine and intra-ovarian alterations establish a self-reinforcing pathogenic loop in which granulosa cells are not passive targets but active mediators of ovarian dysfunction.

**Figure 2:**
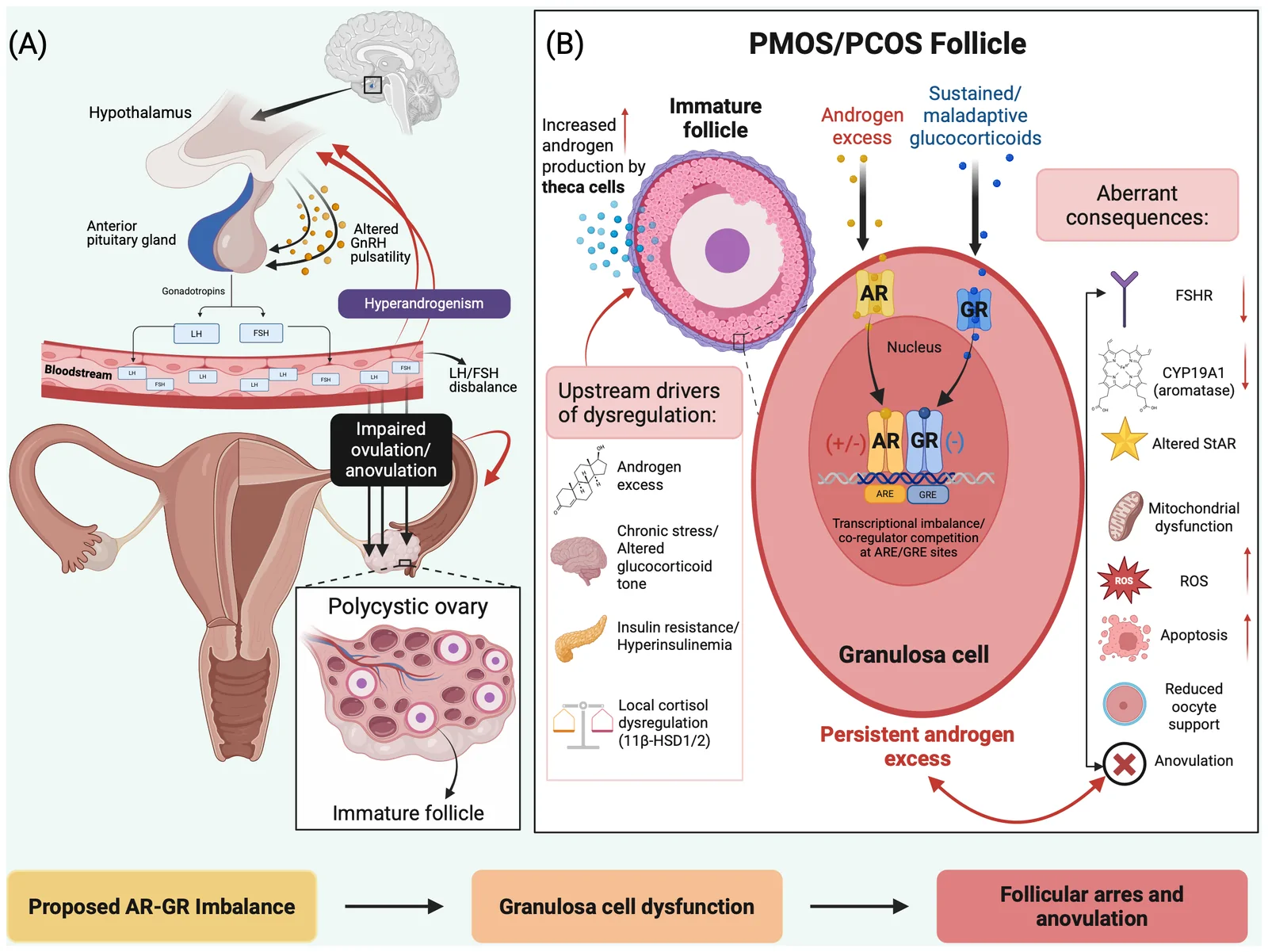
Dysregulated HPO feedback and proposed model of granulosa cell dysfunction in PMOS/PCOS. **(A)** Schematic representation of the dysregulated HPO axis in PMOS. Increased pulsatile secretion of GnRH from the hypothalamus promotes preferential release of LH over FSH by the anterior pituitary. Elevated LH stimulates androgen production by ovarian theca cells, leading to hyperandrogenism, impaired follicular maturation, anovulation, and the accumulation of immature follicles characteristic of polycystic ovaries. **(B)** Proposed mechanisms linking AR and GR signaling to granulosa cell dysfunction. Excess androgens and maladaptive glucocorticoid signaling converge on deregulation of AR and GR respectively, resulting in transcriptional imbalance through interactions at AREs and GREs. The +/− sign indicates context-dependent AR effects, whereas the - sign denotes the predominantly detrimental effects proposed for sustained or maladaptive GR activation. This process may be further amplified by chronic stress, insulin resistance/hyperinsulinemia, and altered local cortisol metabolism mediated by 11β-HSD1/2. The resulting dysregulation of FSHR, CYP19A1, and StAR, together with mitochondrial dysfunction, increased ROS production, apoptosis, and reduced oocyte support, contributes to persistent androgen excess, granulosa cell dysfunction, follicular arrest, and chronic anovulation. PMOS, Polyendocrine metabolic ovarian syndrome; PCOS, polycystic ovary syndrome; HPO, hypothalamic–pituitary–ovarian; GnRH, gonadotropin-releasing hormone; LH, luteinizing hormone; FSH, follicle-stimulating hormone; AR, androgen receptor; GR, glucocorticoid receptor; ARE, androgen response element; GRE, glucocorticoid response element; 11β-HSD1/2, 11β-hydroxysteroid dehydrogenase type 1 and type 2; FSHR, follicle-stimulating hormone receptor; CYP19A1, cytochrome P450 family 19 subfamily A member 1 (aromatase); StAR, steroidogenic acute regulatory protein; ROS, reactive oxygen species. Created in BioRender. Pinna, M (2026). https://BioRender.com/gefpgkm.

## 3 Granulosa cells as central regulators of folliculogenesis

### 3.1 Granulosa-cell control of follicular growth and steroidogenesis

Within the ovarian follicle, GRCs play a central role in coordinating follicular growth, steroidogenesis, and oocyte maturation. By surrounding and supporting the developing oocyte, they coordinate follicular growth through a complex network of endocrine, paracrine, and autocrine signaling pathways while dynamically responding to gonadotropins and local ovarian factors (42).

Throughout folliculogenesis, GRCs undergo extensive proliferation and differentiation as follicles progress from the preantral to the antral stage. Among the endocrine regulators involved in this process, follicle-stimulating hormone (FSH) represents the primary driver of GRC function. Binding of FSH to its cognate receptor activates the cyclic AMP (cAMP)/protein kinase A (PKA) signaling cascade, promoting granulosa-cell proliferation. This cascade also induces the expression of aromatase (CYP19A1), the key enzyme that converts theca-derived androgen precursors into estrogens (40, 43, 44).

Aromatase is the only enzyme in vertebrates capable of catalyzing estrogen biosynthesis from androgens through a multistep oxidative process that ultimately generates estradiol, a hormone indispensable for follicular growth, selection, and reproductive competence (45). During later stages of follicular maturation, LH further modulates granulosa-cell activity by promoting luteinization and the acquisition of progesterone-producing capacity, processes required for ovulation and subsequent corpus luteum formation. Beyond their role in steroidogenesis, GRCs function as critical mediators of intra-follicular communication. They produce and respond to several members of the transforming growth factor-β (TGF-β) superfamily, including activins, inhibins, and AMH, which collectively regulate follicle recruitment, selection, and atresia (46). Among these factors, AMH is secreted primarily by granulosa cells of preantral and small antral follicles and serves as a key regulator of ovarian reserve by limiting excessive follicular recruitment and modulating gonadotropin responsiveness (47).

### 3.2 Granulosa-cell integration of metabolic and stress-related signals

Granulosa cells also integrate systemic metabolic and stress-related signals. In addition to gonadotropins, they respond to metabolic hormones and growth factors such as insulin and insulin-like growth factor 1 (IGF-1), which influence follicular growth and steroidogenic activity (48, 49). Furthermore, GRCs express functional GR and are highly sensitive to glucocorticoid signaling, linking ovarian physiology to systemic stress responses (50). Through the integration of reproductive, metabolic, and endocrine cues, granulosa cells occupy a central position in the regulation of ovarian homeostasis. Consequently, alterations in GRC proliferation, differentiation, steroidogenesis, or hormonal responsiveness can profoundly impair follicular development and contribute to the ovarian dysfunction characteristic of PCOS. Their strategic role at the intersection of multiple signaling pathways makes them particularly vulnerable to disturbances in steroid receptor signaling and supports their relevance as a key cellular target in the pathophysiology of the syndrome.

## 4 Androgen and glucocorticoid signaling in granulosa cells in PCOS

As discussed above, GRCs integrate a broad spectrum of endocrine and metabolic signals to coordinate follicular growth, steroidogenesis, and ovulatory competence. Within this regulatory network, AR and GR signaling represent interconnected pathways through which reproductive, metabolic, and stress-related cues may be integrated. Both receptors belong to the NR3C nuclear receptor family and share regulatory features that provide a molecular basis for potential crosstalk and convergence of their downstream effects (51). Direct molecular evidence of AR-GR interaction, including co-occupancy at shared DNA response elements and competition for transcriptional co-regulators, derives primarily from prostate and hepatic cell models. Comparable data specifically in granulosa cells are currently lacking, and the crosstalk framework discussed below should therefore be regarded as a hypothesis extrapolated to the ovarian context. In PCOS, chronic hyperandrogenism, altered glucocorticoid metabolism, and persistent low-grade inflammation may perturb the physiological balance between these two pathways (52, 53). This hormonal milieu is further shaped by local steroid metabolism: 11β-hydroxysteroid dehydrogenase type 1 and type 2 (11β-HSD1 and 11β-HSD2) regulates local glucocorticoid availability, whereas bioactive androgen metabolites, including 11-oxygenated androgens, add complexity to androgen signaling (54–56). AR-GR interplay should therefore be considered within the broader endocrine and metabolic context of the follicle rather than as an isolated or established receptor interaction. The molecular determinants of this crosstalk and its potential implications for granulosa-cell function are discussed in the following sections.

## 5 Molecular basis of androgen and glucocorticoid receptor crosstalk

The coordinated action of steroid hormones is essential for GRC physiology; however, the ovarian microenvironment is exposed to converging inputs from multiple endocrine axes. A major point of convergence between AR and GR signaling resides in the architecture of their DNA-binding motifs. Both receptors recognize highly similar steroid response elements composed of AGAACA-like half-sites arranged as inverted repeats separated by three nucleotides, commonly referred to as glucocorticoid response elements (GREs) or androgen response elements (AREs) (51). The canonical motif can be represented as AGAACAnnnTGTTCT, although sequence variations and local chromatin context strongly influence receptor occupancy and transcriptional output. In non-ovarian cell systems this structural similarity permits AR and GR to occupy overlapping genomic regions and regulate common target genes. Receptor binding is not determined solely by motif sequence: chromatin accessibility, pioneer transcription factors, histone modifications, and local epigenetic landscapes critically influence receptor recruitment and activity (57, 58). For example, AR binding is frequently facilitated by pioneer factors such as FOXA1, whereas GR recruitment is often favored under stress-associated chromatin states enriched in active enhancer marks. When co-expressed within the same cellular environment, AR and GR may compete for shared response elements, leading to reciprocal modulation of transcriptional programs. Under androgen-rich conditions, AR signaling may dominate the transcriptional landscape and promote gene networks involved in proliferation, metabolism, and steroidogenesis. Conversely, during chronic stress or elevated glucocorticoid exposure, GR activation can redirect chromatin accessibility and reshape transcriptional responses, favoring pathways associated with stress adaptation, metabolic regulation, and growth restraint (51, 59). These key concepts are summarized in **Table 1**. Beyond direct competition for DNA-binding sites, AR and GR also share overlapping co-regulatory networks. Both receptors recruit common transcriptional coactivators, including SRC family proteins, p300/CBP, and SWI/SNF chromatin-remodeling complexes (60). Because these cofactors are available in limited amounts, simultaneous activation of AR and GR may result in functional competition, whereby signaling through one receptor indirectly attenuates transcriptional activity mediated by the other (61). These data provide strong mechanistic plausibility for AR-GR crosstalk even though cistromic evidence derives from prostate or hepatic models rather than primary human granulosa cells. AR–GR crosstalk should not be viewed as a simple competition for DNA occupancy but rather as a multilayered regulatory process integrating receptor abundance, chromatin accessibility, cofactor availability, and endocrine context. In GRCs, whether and how such interactions occur remains unknown. If present, they could influence the integration of reproductive and stress-related signals controlling follicular development, steroidogenesis, and ovarian homeostasis.

**Table 1:** Key concepts and evidence limitations relevant to proposed AR-GR crosstalk in granulosa cells.

Key points
AR and GR recognize similar response elements and recruit overlapping co-regulatory proteins in non-ovarian models. When co-expressed, their transcriptional outputs may overlap, compete, or cooperate depending on chromatin and cellular context. In granulosa cells, androgen excess and altered glucocorticoid tone may converge functionally on proliferation, steroidogenesis, metabolism, and stress responses • This competition is proposed as one mechanistic explanation for why hyperandrogenism and dysregulated cortisol metabolism jointly disrupt follicle development in PCOS.

AR, androgen receptor; GR, glucocorticoid receptor; PCOS, polycystic ovary syndrome.

## 6 Functional consequences of androgen and glucocorticoid receptor crosstalk in granulosa cells

### 6.1 Granulosa-cell fate, metabolic homeostasis, and follicular competence

GRC proliferation is a fundamental determinant of follicular growth and maturation and is tightly regulated by endocrine, paracrine, and metabolic signals. Among steroid hormone receptors, AR signaling plays a key physiological role in supporting GRC proliferation, whereas GR activation often exerts opposing effects, particularly under conditions of chronic stress (62). GRCs express high levels of AR during the early stages of follicular development, and experimental models have demonstrated that granulosa-cell-specific AR deletion results in impaired follicle growth, increased follicular atresia, and reduced fertility, highlighting the importance of androgen signaling in normal ovarian physiology (63). Mechanistically, AR activation enhances GRC responsiveness to FSH by increasing FSH receptor expression and potentiating downstream signaling pathways involved in cell-cycle progression. This includes the regulation of cyclins and other proliferative mediators that facilitate transition through the G1/S checkpoint and sustain follicular growth (12). Conversely, prolonged glucocorticoid exposure has been associated with reduced GRC viability and diminished responsiveness to proliferative stimuli, including FSH and IGF-1, partly through suppression of FSHR and IGF1R expression (64). These findings suggest a dynamic balance between AR-mediated permissive effects and GR-dependent stress-adaptive or growth-restraining mechanisms (50). Disruption of this balance may contribute to impaired follicular development and ovarian dysfunction in PCOS.

Beyond proliferation, AR and GR signaling converge on metabolic pathways that are essential for GRC function and follicular competence. Physiological AR activity supports mitochondrial integrity by promoting mitochondrial biogenesis, maintaining membrane potential, and sustaining ATP production through the regulation of genes involved in oxidative phosphorylation and fatty acid oxidation (65). Experimental models lacking AR activity exhibit reduced mitochondrial DNA content, altered mitochondrial dynamics, and impaired lipid utilization, resulting in compromised cellular bioenergetics and diminished support for oocyte development (66). However, excessive androgen exposure may have detrimental consequences. Hyperandrogenic conditions have been associated with mitochondrial dysfunction, altered electron transport chain activity, increased reactive oxygen species (ROS) generation, and dysregulated lipid metabolism, all of which have been described in experimental models of PCOS (67). Glucocorticoids further influence GRC metabolism, particularly under chronic stress. Sustained GR activation can alter the balance between glycolytic and oxidative pathways, impair mitochondrial efficiency, and promote metabolic adaptations that favor cellular stress responses overgrowth and differentiation (68). In this context, AR and GR signaling exert partially opposing influences on cellular metabolism. While AR generally supports anabolic processes and mitochondrial homeostasis, GR activation promotes catabolic adaptation and metabolic conservation (65, 69). Evidence from tissues co-expressing both receptors suggests that sustained GR activation can redirect or override AR-driven metabolic programs (70). Consequently, AR-GR crosstalk may represent a critical determinant of GRC bioenergetic status, linking endocrine and stress-related signals to ovarian function.

### 6.2 Gonadotropin responsiveness and steroidogenic output

Steroidogenesis within GRCs is tightly regulated by gonadotropin signaling (**Table 2**). FSH promotes aromatase expression and estradiol production, whereas LH drives luteinization and progesterone synthesis during the periovulatory period (81). AR and GR signaling intersect with these pathways at multiple regulatory levels, influencing both the magnitude and quality of gonadotropin responses. Physiological AR signaling enhances GRCs sensitivity to FSH by increasing FSHR expression, potentiating cAMP/PKA signaling, and promoting the transcription of key steroidogenic genes, including CYP19A1, HSD17B1, and STAR (71, 73, 82). Beyond this permissive role, AR has also been implicated in the direct regulation of aromatase expression through cooperation with transcription factors such as FOXL2, thereby influencing intrafollicular estrogen production (74). By contrast, GR activation through sustained glucocorticoid exposure has been associated with impaired ovarian development and reduced steroidogenic output in animal models, while GR-dependent suppression of aromatase has also been demonstrated in a non-ovarian steroidogenic model (75, 83). In addition, elevated glucocorticoid exposure may indirectly impair ovarian steroidogenesis by suppressing LHβ transcription at the pituitary level, thereby reducing luteotrophic support and limiting progesterone production (84). At the molecular level, in prostate and other non-ovarian models, AR and GR can compete for shared transcriptional cofactors and regulatory regions associated with steroidogenic genes. This competition can alter chromatin accessibility and reshape transcriptional responses at loci controlling estradiol and progesterone synthesis, including CYP19A1, HSD17B1, and STAR (58). The net steroidogenic output of GRCs therefore reflects the dynamic balance between androgenic and glucocorticoid signaling. Under physiological conditions, AR activity promotes follicular maturation and steroidogenic competence, whereas sustained GR activation may antagonize these processes and contribute to the impaired follicular development characteristic of PCOS (85, 86).

**Table 2:** AR–GR crosstalk in granulosa cells: molecular mechanisms and functional outcomes in PCOS.

Process	Molecular mechanism			Functional outcomes		
	AR	GR	AR–GR crosstalk	PCOS dysregulation	Clinical outcomes	Ref.
Follicular growth/FSH response	↑ FSHR expression; supports early follicle growth	Modulates stress response and FSH sensitivity	Shared regulation of gonadotropin-responsive genes	Androgen excess; altered GR tone	Follicular arrest; anovulation	(13, 53, 63, 71)
Proliferation and differentiation	↑ proliferation (stage-dependent)	↓ proliferation under chronic stress	Competition for co-regulators	Impaired growth–differentiation balance	Reduced granulosa cell competence	(62–64, 72)
Steroidogenesis	Modulates CYP19A1, STAR, HSD17B1	May suppress gonadotropin-induced steroid production	Convergent control of steroidogenic genes	↓ aromatase activity; ↑ androgenic milieu	Altered estradiol production	(40, 58, 71, 73–75)
Mitochondrial metabolism	Supports bioenergetic activity	Regulates metabolic stress adaptation	Joint impact on oxidative metabolism	Mitochondrial dysfunction, ROS, metabolic stress	Insulin resistance, chronic low-grade inflammation	(66–68, 76, 77)
Apoptosis/stress response	Context-dependent survival and apoptosis	Chronic activation favors apoptosis	Shared stress-response pathways	Persistent endocrine-metabolic stress	↑ apoptosis; impaired follicle viability	(50, 72, 78)
Overall effect	Physiological when balanced	Adaptive when transient	Balance maintains follicular function	Persistent AR–GR imbalance	Anovulation and reduced reproductive competence	(14, 51, 52, 58, 79, 80)

The table summarizes the principal granulosa-cell processes influenced by AR-GR signaling and their crosstalk. The table distinguishes receptor-specific evidence from proposed mechanisms of AR-GR convergence and their possible clinical consequences The left section details molecular mechanisms, whereas the right section highlights their dysregulation in PCOS and the associated clinical consequences.

AR, androgen receptor; GR, glucocorticoid receptor; PCOS, polycystic ovary syndrome; FSH, follicle-stimulating hormone; FSHR, follicle-stimulating hormone receptor; CYP19A1, cytochrome P450 family 19 subfamily A member 1 (aromatase); STAR, steroidogenic acute regulatory protein; HSD17B1, hydroxysteroid 17-beta dehydrogenase 1; ROS, reactive oxygen species.

## 7 Experimental evidence supporting androgen and glucocorticoid receptor interaction in PCOS

### 7.1 *In-vitro* data and animal models

The coordinated activity of steroid receptors is essential for normal ovarian physiology, yet in PCOS GRCs are exposed to a complex and often dysregulated hormonal environment. As previously described, imbalances in AR and GR signaling may disrupt follicular growth, steroidogenesis and stress responses, contributing to the hallmark features of the syndrome such as anovulation, hyperandrogenism and metabolic dysregulation.

Experimental studies combining GRC cultures and animal models of PCOS provide compelling evidence that androgen and glucocorticoid signaling exert tightly interconnected and context-dependent effects on follicular physiology. Under hyperandrogenic conditions, excessive AR activation promotes GRC dysfunction through both apoptotic and metabolic mechanisms. Several studies have demonstrated that androgens increase *Klotho* expression through AR-dependent pathways, thereby promoting GRCs apoptosis and compromising follicular viability (78). In parallel, androgen-induced metabolic stress contributes to cellular dysfunction through lactic acid accumulation, intracellular acidification, and activation of apoptotic signaling cascades (87). More recently, androgen exposure has also been shown to drive NCOA4-dependent ferritinophagy and ferroptotic cell death in human and rodent granulosa cells, identifying iron-dependent cell death as an additional mechanism linking hyperandrogenism to follicular loss; notably, NCOA4 also functions as an AR coregulator, further connecting this pathway to AR signaling (88).

Beyond apoptosis, androgen excess also induces profound metabolic reprogramming. In experimental PCOS models, AR activation enhances SLC1A5-dependent amino acid uptake and alters cellular metabolic pathways, supporting abnormal granulosa-cell function while impairing normal follicular differentiation (76). Importantly, the biological consequences of AR activation are highly dependent on the surrounding endocrine environment. Experimental evidence indicates that physiological androgen signaling can partially rescue ovarian follicle function impaired by excessive glucocorticoid exposure through synergistic interactions with IGF-1 and FSH signaling pathways (53). These findings support the concept that AR activity may exert beneficial effects under physiological conditions but becomes detrimental when excessively activated. Dysregulated glucocorticoid signaling also contributes significantly to PCOS-associated ovarian and metabolic dysfunction. Pharmacological inhibition of GR signaling improves glucose metabolism and systemic metabolic parameters in hyperandrogenic animal models, supporting a pathogenic role for excessive glucocorticoid activity (89). Additional signaling pathways appear to integrate androgenic and glucocorticoid stimuli within GRCs. Among these, WNT5A-mediated activation of the PI3K/AKT pathway has been implicated in impaired steroidogenesis, reduced proliferation, and increased apoptosis in PCOS granulosa cells (72). Given that both AR and GR signaling converge on PI3K/AKT regulation, this pathway may represent an important molecular hub linking endocrine dysfunction to follicular pathology. Collectively, experimental evidence supports a model in which physiological AR signaling promotes follicular competence, whereas androgen excess combined with dysregulated glucocorticoid signaling drives metabolic stress, apoptosis, and follicular arrest, thereby contributing to the ovarian phenotype characteristic of PCOS.

### 7.2 Human evidence and translational relevance

Clinical and patient-derived studies provide strong support for the relevance of AR–GR crosstalk in PCOS and highlight the importance of local glucocorticoid metabolism in regulating GRC function. Evidence of glucocorticoid resistance observed across different stages of life in women with PCOS suggests that altered GR responsiveness may further exacerbate ovarian dysfunction (90). A central component of this regulatory network is represented by 11β-HSD (91). Within the follicle, 11β-HSD1 primarily regenerates active cortisol from cortisone, whereas 11β-HSD2 inactivates cortisol; net local glucocorticoid availability depends on their relative expression and activity (91). Increased 11β-HSD1 activity has been associated with insulin resistance in granulosa cells obtained from women with PCOS, linking altered glucocorticoid metabolism to local metabolic dysfunction (79). Additional studies have suggested that glucocorticoids and 11β-HSD enzymes play broader roles in coordinating ovarian steroidogenesis and follicular development (92), suggesting that local cortisol exposure may negatively affect follicular competence and reproductive success (80). The functional relevance of local glucocorticoid metabolism is further supported by studies demonstrating direct inhibitory effects of cortisol on ovarian steroidogenesis, with 11β-HSD enzymes acting as key regulators of glucocorticoid bioavailability within the ovary (80). Human luteinized GRCs express 11β-HSD1 transcripts and therefore constitute an active site of intra-ovarian glucocorticoid metabolism (93). Interestingly, ovarian 11β-HSD activity appears to be reduced in women with anovulatory PCOS, potentially as a consequence of chronic androgen excess, suggesting a reciprocal relationship between androgenic and glucocorticoid pathways within the ovary (13). In parallel, multiple studies have documented profound alterations in steroidogenic gene expression and enzyme activity in granulosa cells from women with PCOS (94, 95). Transcriptomic analyses have identified dysregulated pathways involved in steroid biosynthesis, metabolic regulation, and hormone responsiveness, indicating that GRC dysfunction persists even outside the ovarian environment (95). Furthermore, insulin resistance appears to modulate these molecular alterations, as distinct gene-expression profiles have been described in granulosa cells from women with PCOS with or without metabolic impairment (96). Emerging evidence also suggests that altered communication between granulosa and theca cells may amplify steroid receptor dysregulation. Recent studies identifying distinct theca-cell subpopulations support the concept that abnormal paracrine interactions contribute to the endocrine abnormalities observed in PCOS (97, 98). Overall, patient-derived evidence supports a model in which androgen excess, dysregulated glucocorticoid metabolism, altered cortisol availability, and aberrant steroidogenic signaling converge to disrupt granulosa-cell homeostasis. These observations provide clinically relevant support for a role of AR-GR imbalance in the pathophysiology of PCOS.

## 8 Biomarker and therapeutic implications

### 8.1 Circulating and follicular biomarkers of steroid receptor imbalance

These experimental and patient-derived findings highlight the clinical relevance of dysregulated steroid receptor signaling in PCOS. From a therapeutic standpoint, targeting pathways that modulate AR–GR signaling may represent a promising strategy to restore follicular competence. Pharmacological approaches aimed at reducing androgen excess, modulating GR activity, or correcting local steroid metabolism could potentially improve granulosa-cell function and ovarian responsiveness. Moreover, interventions targeting metabolic and stress-related pathways, such as insulin-sensitizing therapies or strategies that normalize hypothalamic–pituitary axis activity, may indirectly restore the physiological balance between androgenic and glucocorticoid signaling within the ovary (99). Increasing attention has focused on the diagnostic and biomarker potential of steroid-signaling pathways involved in PCOS. As discussed previously, 11-oxygenated androgens constitute a major component of the circulating androgen pool in women with PCOS and may better reflect androgenic activity than conventional androgens in selected patient populations (21). In parallel, alterations in local glucocorticoid metabolism within the ovary, have been associated with changes in follicular steroidogenesis and metabolic function, suggesting that intra-ovarian cortisol availability may directly influence granulosa-cell physiology and reproductive outcomes (100). The follicular microenvironment itself represents an additional source of potential biomarkers. Molecular characterization of granulosa cells obtained during assisted-reproduction procedures has revealed alterations in steroidogenic enzymes, receptor-signaling pathways, and metabolic networks in PCOS follicles (36). These findings raise the possibility that integrated profiling of androgenic and glucocorticoid signaling, combining circulating steroid measurements with follicular biomarkers, could improve patient stratification and provide a more refined understanding of the heterogeneous endocrine landscape of PCOS. Altogether, integrating molecular insights into steroid receptor crosstalk with clinical and metabolic features of the syndrome may ultimately facilitate the development of biomarker-guided approaches aimed at improving diagnosis, reproductive management, and long-term health outcomes in women with PCOS.

### 8.2 Current and emerging therapeutic strategies

Building on these emerging molecular insights, therapeutic strategies for PCOS are increasingly being interpreted within the broader framework of endocrine and metabolic dysregulation. Current clinical management primarily focuses on alleviating the main manifestations of the syndrome, including hyperandrogenism, ovulatory dysfunction, and metabolic abnormalities (7). Combined oral contraceptives remain the first-line pharmacological option for women not seeking pregnancy, partly because of their antiandrogenic effects (101). By suppressing gonadotropin secretion through inhibition of the HPO axis and increasing circulating sex hormone-binding globulin levels, these treatments reduce ovarian androgen production and decrease androgen bioavailability. Although they do not directly target the AR–GR axis, their net effect is to lower androgen exposure and thereby indirectly reduce AR stimulation in target tissues, including the ovary. In selected cases, antiandrogenic agents such as spironolactone (102) or cyproterone acetate (103) may be used as adjunctive therapies to directly antagonize AR signaling. These drugs act downstream of the AR–GR crosstalk by blocking AR activation. Because metabolic disturbances represent a central component of PCOS pathophysiology, insulin-sensitizing therapies are widely employed in clinical practice. Among these, metformin has been extensively investigated and has been shown to improve insulin sensitivity, reduce circulating insulin concentrations, and indirectly attenuate ovarian androgen production (99). These effects may secondarily influence AR signaling by lowering substrate availability for androgen synthesis, but a direct action on the AR–GR axis has not been demonstrated. More recently, incretin-based therapies such as GLP-1 receptor agonists, including liraglutide and semaglutide, have attracted considerable attention because of their beneficial effects on weight loss, glucose homeostasis, metabolic risk profiles, and ovulatory function (104). Similarly, SGLT-2 inhibitors, such as empagliflozin, are being explored as potential adjunctive treatments for metabolic dysfunction, although evidence in PCOS remains limited (105). To date, there is no direct evidence that GLP-1 receptor agonists or SGLT-2 inhibitors modulate AR or GR signaling in the ovary; their benefits appear to be mediated mainly through systemic metabolic improvement. Lifestyle interventions, including dietary modification and regular physical activity, remain a cornerstone of PCOS management because they improve insulin resistance, endocrine balance, and reproductive outcomes (106). These interventions may also indirectly affect AR–GR signaling by reducing systemic inflammation, oxidative stress, and hyperinsulinemia, but specific data on AR–GR modulation in PCOS are lacking. Despite these available therapeutic options, most current interventions target downstream manifestations of the syndrome rather than the molecular mechanisms underlying disease development. In this context, the growing body of evidence supporting functional crosstalk between AR and GR suggests that modulation of glucocorticoid signaling and local steroid metabolism may represent an additional and largely unexplored therapeutic avenue (77). Specifically, modulation of glucocorticoid signaling and metabolism, as well as direct targeting of GR activity, remain speculative therapeutic strategies in PCOS and are not currently approved for clinical use. Potential interventions acting on GR or cortisol metabolism could have broad effects on the HPA axis, immune function, endometrium, ovulation, pregnancy, and cardiometabolic homeostasis (54, 89). Speculative therapeutic avenues could include selective glucocorticoid receptor modulators that may dissociate beneficial metabolic effects from adverse reproductive outcomes, 11β-HSD antagonists to reduce local cortisol regeneration within the ovary, an approach for which proof-of-concept dissociation of glucocorticoid metabolic effects has been demonstrated in a clinical trial setting (107), and combination approaches targeting both AR and GR signaling alongside metabolic interventions. To date, these approaches should be considered hypothetical and require dedicated mechanistic and clinical studies. A deeper understanding of AR–GR interactions within the ovarian microenvironment could therefore contribute to the development of more targeted and personalized therapeutic strategies for women with PCOS.

## 9 Conclusions and further directions

PCOS is a complex endocrine and metabolic disorder characterized by the interplay of reproductive, metabolic, inflammatory and neuroendocrine abnormalities. Hyperandrogenism is a central driver of disease pathophysiology, while glucocorticoid signaling and local cortisol metabolism may also contribute to ovarian dysfunction. The molecular mechanisms underlying granulosa-cell dysfunction remain incompletely understood. AR and GR signaling, and their potential interplay, should therefore be viewed as one component of a broader pathogenic network, with possible implications for future biomarker discovery and mechanism-based research.

GRCs occupy a central position within this regulatory network, integrating gonadotropin, metabolic, androgenic, and glucocorticoid signals that collectively determine follicular development and steroidogenic competence. The molecular interactions between AR and GR extend beyond simple receptor activation: they involve shared response elements, common co-regulators, chromatin remodeling mechanisms, and overlapping transcriptional programs. Experimental, translational, and patient-derived studies collectively support the existence of functionally relevant AR–GR crosstalk within the ovarian microenvironment, and disruption of this balance may contribute to altered steroidogenesis, impaired follicular maturation, metabolic dysfunction, and reduced reproductive competence in women with PCOS.

Although important mechanistic questions remain unresolved, the emerging concept of AR–GR interaction offers a novel framework for understanding ovarian dysfunction in PCOS. Future work should combine LC-MS/MS steroid profiling, follicular-fluid cortisol/cortisone assessment, single-cell or sorted-cell transcriptomics, chromatin-accessibility mapping, receptor-occupancy studies, and functional AR/GR perturbation in patient-derived granulosa cells. Such studies will be essential to determine whether AR–GR imbalance can support biomarker-based stratification or targeted therapeutic development in PCOS. Candidate therapeutic strategies, still preliminary and untested, might include selective GR modulation, ovarian inhibition of cortisol regeneration, or combined AR–GR targeting in carefully selected phenotypes, potentially guided by biomarkers such as circulating 11-oxygenated androgens, follicular fluid cortisol/cortisone ratios, or granulosa-cell AR/GR expression profiles.

Finally, it is tempting to speculate that distinct patterns of receptor crosstalk may underlie the clinical heterogeneity of the syndrome. AR-predominant signaling, sustained by high circulating and intra-ovarian androgens including 11-oxygenated androgens, may preferentially characterize phenotypes dominated by hyperandrogenism and oligo-anovulation. A relative shift toward GR-driven signaling, potentially reflecting chronic stress and altered local cortisol metabolism, could instead contribute more to the metabolic and insulin-resistant features seen in other phenotypic presentations, while milder or more balanced AR–GR crosstalk might be compatible with phenotypes in which granulosa-cell dysfunction and hyperandrogenism are less pronounced. This model is hypothetical and requires studies correlating receptor expression and activity in GRCs with rigorously defined clinical and biochemical phenotypes.
